# Deep Sequencing of Urine Specimens Detects Two BK Polyomavirus Genotypes in a Hematopoietic Stem Cell Transplant Recipient

**DOI:** 10.1128/MRA.01461-19

**Published:** 2020-01-30

**Authors:** Trang Dinh Van, Dominic E. Dwyer, Neisha Jeoffreys, Jen Kok, Brian J. Nankivell, Rebecca J. Rockett

**Affiliations:** a Clinical Laboratory, National Hospital of Tropical Diseases, Dong Da, Hanoi, Vietnam; b Sydney Medical School, The University of Sydney, Camperdown, NSW, Australia; c Centre for Infectious Diseases and Microbiology-Public Health, Sydney, NSW, Australia; d NSW Health Pathology-Institute of Clinical Pathology and Medical Research, Westmead, NSW, Australia; e Department of Renal Medicine, Westmead Hospital, Westmead, NSW, Australia; University of Rochester School of Medicine and Dentistry

## Abstract

BK polyomavirus (BKPyV) is an important pathogen in transplant recipients. We report four draft BKPyV genomes, three of BKPyV genotype I (subtype I-b2) (AUS-105, AUS-106, and AUS-108) and one of genotype II (AUS-107). These draft genomes were identified in longitudinal urine samples collected from a single hematopoietic stem cell transplant recipient.

## ANNOUNCEMENT

BK polyomavirus (BKPyV) is a circular double-stranded DNA virus that belongs to the *Betapolyomavirus* genus of the *Polyomaviridae* family and can cause severe clinical syndromes in renal transplant and hematopoietic stem cell transplant (HSCT) recipients (1, 2). Evidence suggests that *de novo* infection with BKPyV from the donor kidney increases the risk of developing BKPyV-associated disease after transplantation, particularly when the recipient and donor do not harbor the same BKPyV genotype (3). We report the draft genome sequences of two different BKPyV strains infecting a single HSCT recipient, which were detected over a period of 4 months (genotype I [subtype I-b2] and genotype II).

Urine samples were collected on days 12 (AUS-105), 65 (AUS-106), 69 (AUS-107), and 88 (AUS-108) posttransplantation. Viral DNA was extracted from 250 μl of urine using the NucliSENS easyMAG total nucleic acid extraction system (bioMérieux, France), and BKPyV DNA was detected by the BKPyV real-time PCR (RT-PCR) assay described by Hirsch et al., using SensiFAST No-ROX master mix (Bioline, Australia) (4, 5). To enrich BKPyV DNA sequences in the clinical specimens, a directed rolling-circle amplification (dRCA) method was used to generate sufficient BKPyV-specific whole-genome sequencing (WGS) reads (6). After enrichment, DNA libraries were prepared by employing the Nextera XT library preparation kit and subsequently were sequenced by using paired-end 150-bp chemistry on a NextSeq 500 system (Illumina, Australia).

**FIG 1:**
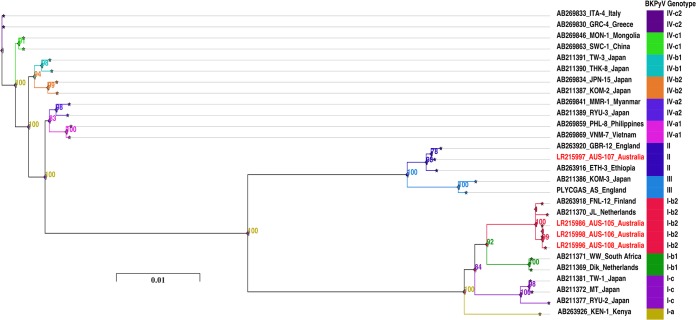
Unrooted maximum likelihood phylogenetic tree of BKPyV genome sequences. The phylogenetic tree was based on the complete consensus sequences of 4 BKPyV-positive samples (AUS-105, AUS-106, AUS-107, and AUS-108) from a single Australian HSCT recipient and 24 BKPyV reference genomes, which are annotated with GenBank accession numbers. The maximum likelihood tree was created using IQ-TREE version 1.6.7 (11) with ModelFinder (best substitution model, general time reversible with empirical base frequencies and rate heterogeneity, allowing for a proportion of invariable sites [GTR+F+I]; number of ultrafast bootstrap replicates, 1,000). The phylogeny was annotated and visualized with Microreact and iTOL (https://itol.embl.de) (12). Genotypes and subtypes are shown on the right in different colors. The headers indicate the GenBank accession number, strain, sample, or isolate name, and country of origin (for example, AB269833_ITA-4_Italy).

Raw sequencing reads were trimmed with Trimmomatic version 0.36, using a sliding window approach with a minimum Phred quality score of 20 (7). Human and microbiome reads were removed by mapping reads to the BKPyV reference genome (GenBank accession number AB263918), using Burrows-Wheeler alignment (default parameters version 0.7.12) (8). Mapped reads were collated and converted to fastq files using SAMtools version 1.6 (AUS-105, 1,559,526 BKPyV-specific reads [75.7% BKPyV-specific reads, 2,060,018 total reads]; AUS-106, 4,885,376 BKPyV-specific reads [98.9% BKPyV-specific reads, 4,939,866 total reads]; AUS-107, 2,767,214 BKPyV-specific reads [98% BKPyV-specific reads, 2,824,324 total reads]; AUS-108, 3,943,064 BKPyV-specific reads [85.5% BKPyV-specific reads, 4,611,286 total reads]) (9). Visualization, alignment, and generation of consensus genomes were conducted using the software package CLC Genomics Workbench version 9.0 (Qiagen, Denmark). The vast majority of reads mapped to the BKPyV reference genome, which resulted in high average mapped read depths for the four samples (AUS-105, 32,204×; AUS-106, 131,191×; AUS-107, 60,615×; AUS-108, 88,633×). The resulting draft BKPyV genomes had GC contents of 39.31% and final lengths of 5,142 bp (AUS-105, AUS-106, and AUS-108) and 5,129 bp (AUS-107). A maximum likelihood phylogenetic tree was constructed from the 4 consensus BKPyV WGS sequences and 24 BKPyV reference sequences from GenBank, which were representative of the main BKPyV genotype/subtype lineages (Fig. 1) (10–12). Samples AUS-105, AUS-106, and AUS-108 were assigned to genotype I (subtype I-b2) and sample AUS-107 to genotype II, because they were closest to the reference strains (GenBank accession numbers AB263918 and AB263920, respectively). We confirmed the observation of two different BKPyV genotypes/subtypes in a single patient by repeating all procedures of DNA extraction, RT-PCR, dRCA, WGS, and phylogenetic analyses from the original clinical urine specimens; the same results were obtained.

This study showed that two BKPyV genotypes were identified in a single patient within 88 days after transplantation. Genotype I (subtype I-b2) was initially present and was replaced by genotype II, which in turn was replaced by the original genotype I (subtype I-b2).

### Data availability.

Fastq files containing BKPyV-specific reads (accession numbers ERR3503274, ERR3503321, ERR3503322, and ERR3503323) and draft BKPyV genome assemblies (accession numbers LR215986, LR215998, LR215997, and LR215996) have been deposited in the European Nucleotide Archive under project number PRJEB29464.
